# Supplementary material for: MK2 deficiency decreases mortality in male mice during the inflammatory phase after myocardial infarction
Source: Physiol Rep. 2025 Sep 19;13(18):e70558. doi: 10.14814/phy2.70558 (PMC12447013; doi:10.14814/phy2.70558)
Supplement: Supplementary file 1 — Figure S1. [file PHY2-13-e70558-s005.zip › PHYSREP-2025-02-099-T-f14-z-.docx]

**Figure S1.** **MK2 deficiency did not hinder recruitment of neutrophils and monocytes to the peri-infarct and infarct regions 3- and 5-days post-MI.**  Representative images of immunohistochemical staining for myeloperoxidase (MPO, dark brown), a neutrophil and monocyte marker, in sham and infarcted hearts from MK2^+/+^ and MK2^-/-^ mice euthanized 3- and 5-days post-MI. Hearts were cut along the short axis through the center of the infarct to yield upper (Section A) and lower, (Section B) regions of the infarct. Bar = 250 μm.
