# Supplementary figures and images for: MK2 deficiency decreases mortality in male mice during the inflammatory phase after myocardial infarction
Source: Physiol Rep. 2025 Sep 19;13(18):e70558. doi: 10.14814/phy2.70558 (PMC12447013; doi:10.14814/phy2.70558)

Supplemental Figure 1

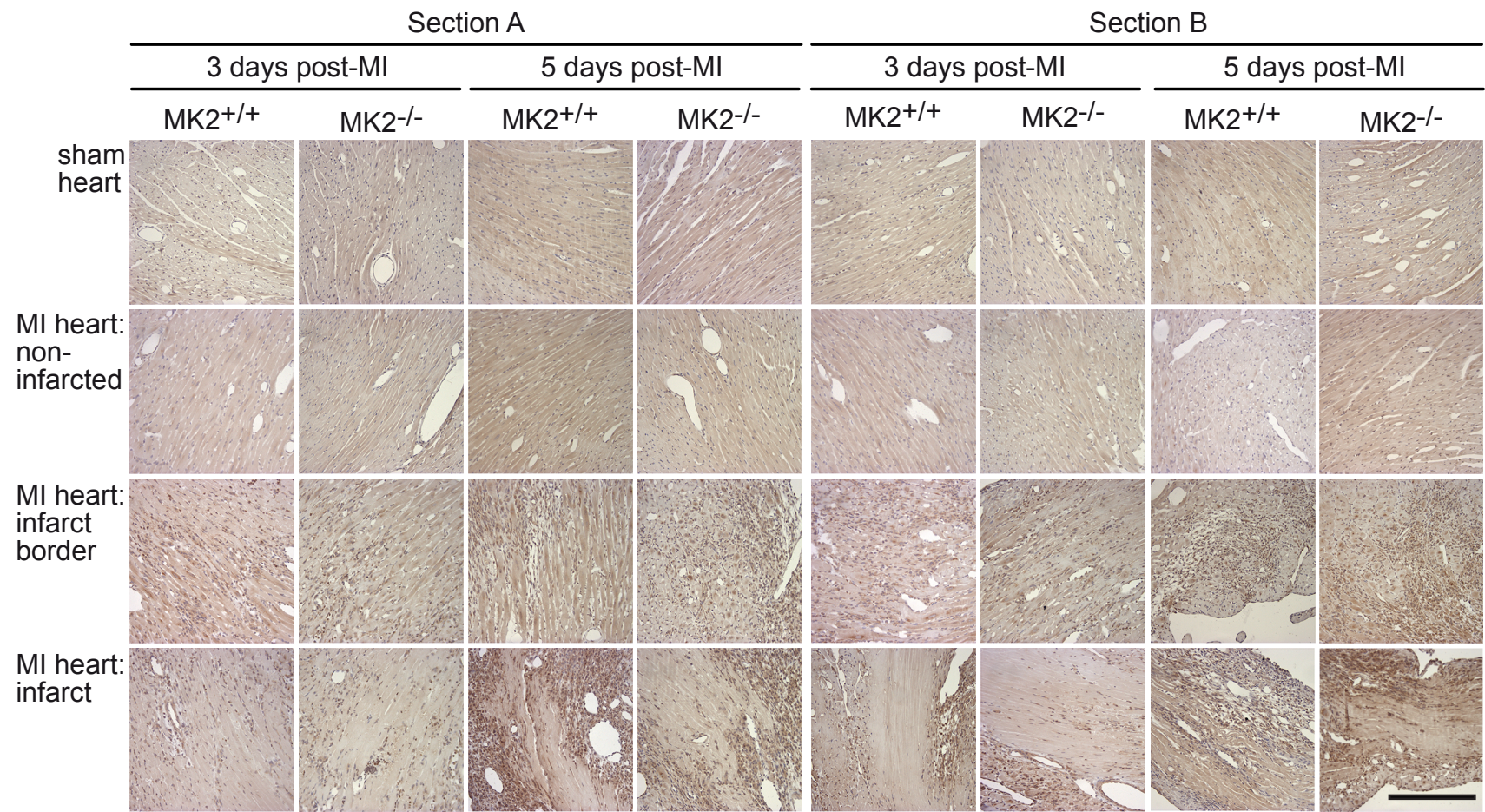

Supplement: Supplementary file 1 — Figure S1. [file PHY2-13-e70558-s005.zip › PHYSREP-2025-02-099-T-f15-z-.pdf]

Supplemental Figure 2

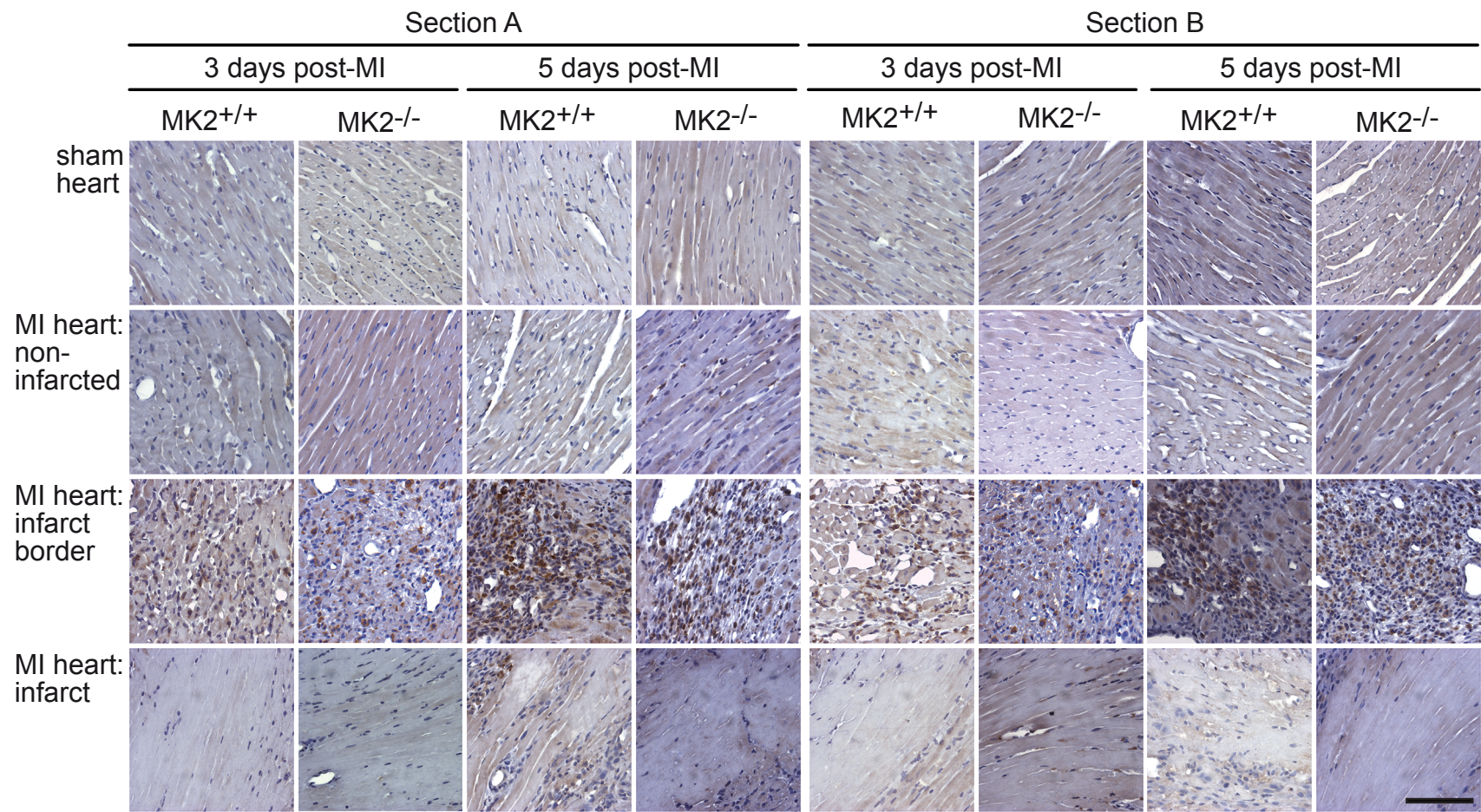

Supplement: Supplementary file 2 — Figure S2. [file PHY2-13-e70558-s020.zip › phy270558-sup-0002-FigureS2rev/Figure S2.pdf]

Supplemental Figure 3

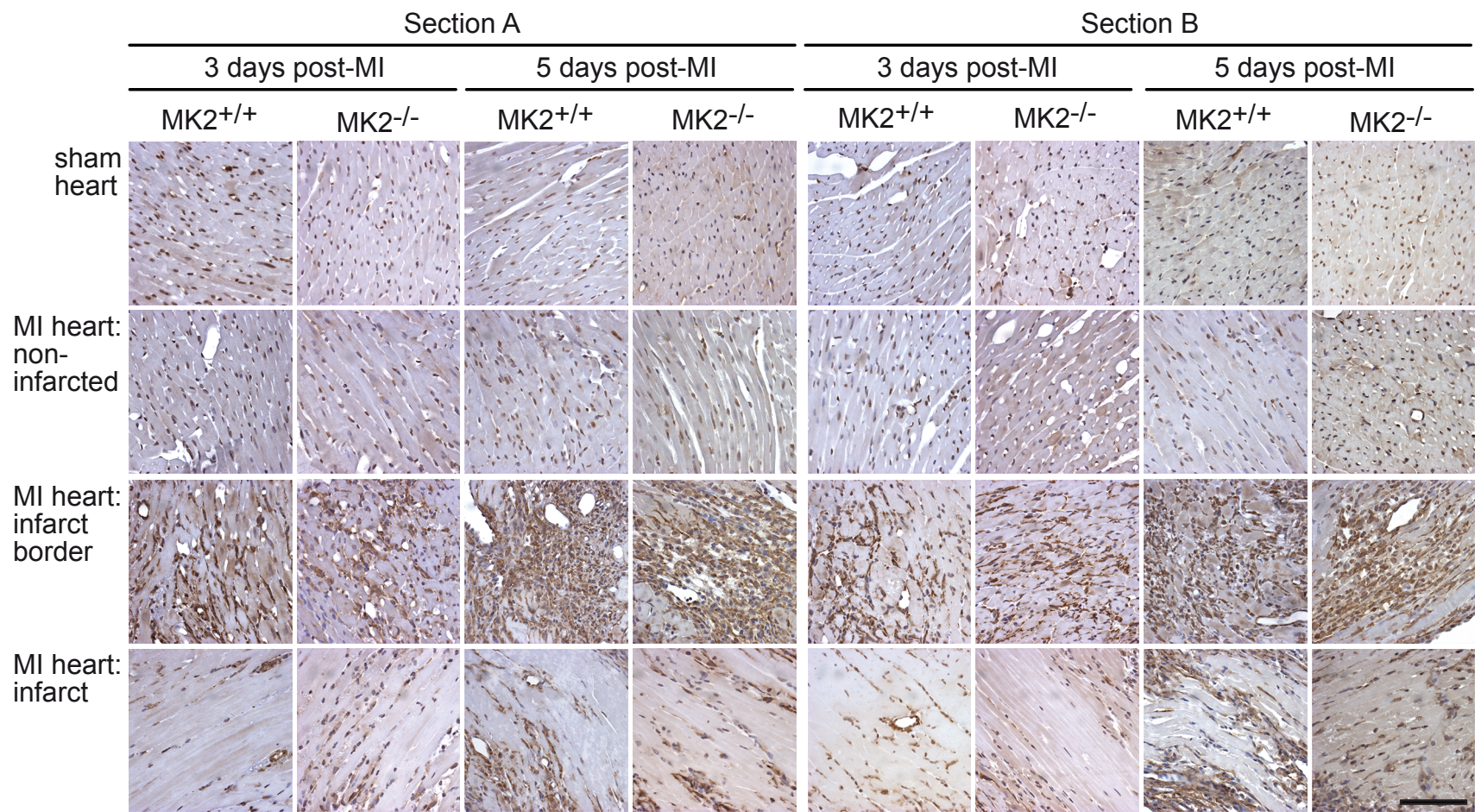

Supplement: Supplementary file 3 — Figure S3. [file PHY2-13-e70558-s014.zip › PHYSREP-2025-02-099-T-f17-z-.pdf]

Supplemental Figure 4

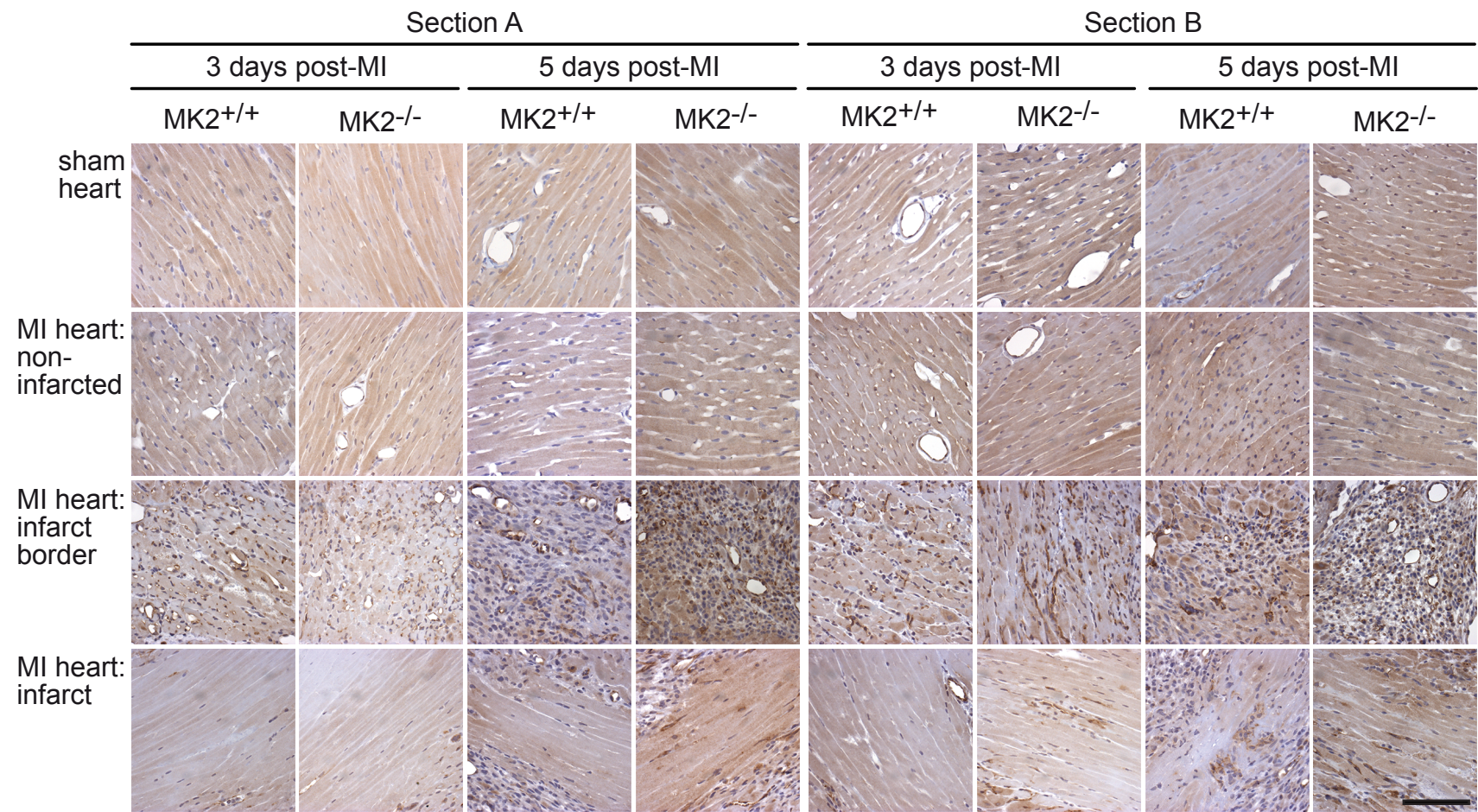

Supplement: Supplementary file 4 — Figure S4. [file PHY2-13-e70558-s010.zip › PHYSREP-2025-02-099-T-f18-z-.pdf]
