# Supplementary material for: MK2 deficiency decreases mortality in male mice during the inflammatory phase after myocardial infarction
Source: Physiol Rep. 2025 Sep 19;13(18):e70558. doi: 10.14814/phy2.70558 (PMC12447013; doi:10.14814/phy2.70558)
Supplement: Supplementary file 2 — Figure S2. [file PHY2-13-e70558-s020.zip › phy270558-sup-0002-FigureS2rev/Figure S2 Legend.docx]

**Figure S2. MK2 deficiency did not hinder M2 macrophage recruitment to the peri-infarct region 3- and 5-days post-MI.**  Representative images of immunohistochemical staining of the mannose receptor cluster of differentiation 206 (CD206, dark brown), an M2 macrophage marker, in sham and infarcted hearts from MK2^+/+^ and MK2^-/-^ mice euthanized 3- and 5-days post-MI. Hearts were cut along the short axis through the center of the infarct to yield upper (Section A) and lower, (Section B) regions of the infarct. Bar = 100 μm.
