# Supplementary material for: MK2 deficiency decreases mortality in male mice during the inflammatory phase after myocardial infarction
Source: Physiol Rep. 2025 Sep 19;13(18):e70558. doi: 10.14814/phy2.70558 (PMC12447013; doi:10.14814/phy2.70558)
Supplement: Supplementary file 3 — Figure S3. [file PHY2-13-e70558-s014.zip › Figure S3.docx]

**Figure S3.** **MK2 deficiency did not alter the distribution or abundance of myofibroblasts 3- and 5-days post-MI.** Representative images of immunohistochemical staining of smooth muscle alpha-actin (α-SMA, dark brown), a myofibroblast marker, in sham and infarcted hearts from MK2^+/+^ and MK2^-/-^ mice sacrificed 3- and 5-days post-MI. Hearts were cut along the short axis through the center of the infarct to yield upper (Section A) and lower, (Section B) regions of the infarct. Bar = 100 μm.
