# Supplementary material for: MK2 deficiency decreases mortality in male mice during the inflammatory phase after myocardial infarction
Source: Physiol Rep. 2025 Sep 19;13(18):e70558. doi: 10.14814/phy2.70558 (PMC12447013; doi:10.14814/phy2.70558)
Supplement: Supplementary file 4 — Figure S4. [file PHY2-13-e70558-s010.zip › Figure S4.docx]

**Figure S4. MK2-deficient hearts showed a greater increase in endothelial cell abundance in their peri-infarct region 5 days post-MI. A**, Representative images of immunohistochemical staining of the cluster of differentiation 31 protein (CD31, dark brown), an endothelial cell marker, in MK2^+/+^ and MK2^-/-^ sham and infarct hearts collected 3- and 5-days post-MI. Hearts were cut along the short axis through the center of the infarct to yield upper (Section A) and lower, (Section B) regions of the infarct. Bar = 100 μm.
